# Prevalence and Correlates of Physical Inactivity among Older Adults in Rio Grande do Sul, Brazil

**DOI:** 10.1371/journal.pone.0117060

**Published:** 2015-02-20

**Authors:** Adelle M. R. Souza, Gerda G. Fillenbaum, Sergio L. Blay

**Affiliations:** 1 Department of Psychiatry, Federal University of São Paulo, (Escola Paulista de Medicina—UNIFESP), São Paulo, São Paulo, Brazil; 2 Center for the Study of Aging and Human Development, Duke University Medical Center, Durham, NC, United States of America; West Virginia University, UNITED STATES

## Abstract

**Background:**

Current information on the epidemiology of physical inactivity among older adults is lacking, making it difficult to target the inactive and to plan for interventions to ameliorate adverse effects.

**Objectives:**

To present statewide representative findings on the prevalence of physical inactivity among older community residents, its correlates and associated health service use.

**Methods:**

A representative non-institutionalized random sample of 6963 individuals in Rio Grande do Sul, Brazil, aged ≥60 years, was interviewed face-to-face. Information was obtained on demographic characteristics, social resources, health conditions and behaviors, health service use, and physical inactivity. Controlled logistic regression was used to determine the association of physical inactivity with these characteristics.

**Results:**

Overall, 62% reported no regular physical activity. Physical inactivity was significantly more prevalent among women, older persons, those with lower education and income, Afro-Brazilians (73%; White: 61%; “other”: 64%), those no longer married, and was associated with multiple individual health conditions and impaired activities of daily living (ADL). In adjusted analyses, associations remained for sociodemographic characteristics, social participation, impaired self-rated health, ADL, vision, and depression (odds ratios (OR) 1.2–1.7). Physically inactive respondents were less likely to report outpatient visits (OR 0.81), but more likely to be hospitalized (OR 1.41).

**Conclusions:**

Physical inactivity is highly prevalent, particularly among Afro -Brazilians. It is associated with adverse sociodemographic characteristics; lack of social interaction; and poor self-rated health, ADL, vision, and depression; although not with other health conditions. Self-care may be neglected, resulting in hospitalization.

## Introduction

The adverse effects of physical inactivity are increasingly recognized, but the correlates of such inactivity in older persons (who are typically less active than those who are younger), particularly those in a middle income country, are less well known, so that awareness of where to intervene remains unclear. The present study is designed to provide information in this area.

In Brazil, older adults are the fastest growing cohort of the population, a trend observed in other developing and industrialized nations [1]. Currently, over 11% of the Brazilian population is aged 60 and over, a proportion expected to increase as the demographic transition progresses [2].

Physical inactivity is increasing rapidly worldwide, with substantial variability across regions, and as a function of gender and age [3]. This is particularly so in China and Brazil, which have the two highest absolute and relative rates of decline in total physical activity, and some of the higher increases in sedentary time [4]. Physical inactivity is a recognized risk factor for health, having been found to be associated with a wide range of health problems particularly prevalent in the elderly, including type 2 diabetes, coronary heart disease, colon and breast cancer, all cause mortality, and premature death [5–9]. There are indications that physical inactivity disproportionately affects ethnic minorities (in particular those of African descent), women and lower socioeconomic groups [10]. In line with these findings, regular physical activity is associated with risk reduction of diseases and of all cause mortality [6,11]. Other benefits include relief of symptoms of depression [12], and increased perception of wellbeing, self confidence and better quality of life [13].

Within European Union countries, 80% of respondents aged 65 years and over reported no vigorous physical activity in the previous 7 days [14]. In Brazil, while only one study to date appears to have focused on the elderly [15], a growing body of research has shown that over 70% of older people are physically inactive, particularly older women, and those of lower socioeconomic status [16–19]. These are the populations that are also more likely to have chronic health conditions. Given the association between physical inactivity and chronic health conditions, physical inactivity may contribute to increased use of the health care system.

Given the negative impact of physical inactivity, data on its prevalence and correlates are needed in order to determine the extent and to whom interventions should be proposed. Presently, several aspects of the epidemiology of physical inactivity are unknown, in part because of the limited sample sizes available to date, and restricted inquiry. First, we need information on the prevalence of physical inactivity in older people, in particular minority and socioeconomically disadvantaged groups. Second, we need information on the association of physical inactivity with health conditions, self-rated health, ability to perform activities of daily living, and specific mental disorders such as depression. Third, it is critical to understand the association with health service utilization.

The present study was designed to provide this information for a middle income country—Brazil—using data from a comprehensive statewide survey: “The Elderly of Rio Grande do Sul”, supported by the State Council on Aging [20]. The richness, representativeness, and size of this sample allow us to examine physical inactivity and its correlates, including sociodemographic characteristics, health behaviors and conditions, and health service use in an older population, including a substantial minority sample.

## Methods

### Study design and sample

The survey “The Elderly of Rio Grande do Sul”, supported by the State Council on Aging [20] focused on adults aged 60 years old and over who lived in private households in the state of Rio Grande do Sul, Brazil. Briefly, this cross-sectional study of noninstitutionalized persons was based on a multistage stratified random sample. The first step was to stratify the 333 municipalities in the state into five categories according to basic economic activity and number of inhabitants. The proportion of each category in each homogeneous zone was calculated, and the number of subjects in each stratum needed to obtain a representative proportion of elderly community residents was determined. Second, the municipalities were randomly selected proportionally in each stratum. The third step was to obtain a random sample of urban census areas for each municipality as supplied by the Brazil Geography and Statistics Institute (Instituto Brasileiro de Geografia e Estatística). Fourth, to get a sample of private households from each of these census areas, a block was randomly selected, and every eighth house was systematically visited in person by the interviewer. If a household had more than one age-eligible person, the respondent was randomly selected. Houses with no eligible person were replaced by the next neighbor.

Trained lay interviewers carried out face-to-face computer-assisted structured interviews on 7040 individuals in eight of the nine regions into which the state was divided (information from one region had to be discarded because of data entry problems). The response rate was 99%, yielding a sample of 6,963 subjects, 6,924 (99.4%) of whom provided information on physical inactivity. All subjects gave oral consent. Recruitment and consent procedures were approved by the ethics committee of the Federal University of São Paulo which specifically approved this study. Details of the survey methodology have been published previously [21].

### Measures

**Physical inactivity**. The specific question that determined physical inactivity was: “In the last three months did you engage in any kind of regular physical activity?” All participants were asked to name the type of physical activity (for example walking, swimming, cycling, workout, etc.), and to indicate how often they practiced the chosen activities, providing less room for ambiguity. Absence of participation indicated physical inactivity. As there was no statistically significant difference in gender or age between those reporting ‘No’ and ‘Don’t know’ both were noted as physically inactive (coded 1), physical activity once or more a week was coded 0 (i.e., not physically inactive).

**Demographic Variables**. Interviewers asked participants basic questions about their sociodemographic background, including age, gender, education (<4 years, ≥4 years; the cut-point reflects the number of years of basic education in Brazil for people of this age), income (<$US200/month [low income] *vs*. ≥$US200/month [higher income]), race/ethnicity (White, Afro-Brazilian, other).

**Social ties and health behaviors**. Social ties and health behaviors included marital status (married, never married, no longer married [divorced, separated, widowed]); report of any current participation in social activities (participation in any formal association on a list that included cultural, sports, recreational, religious, charitable/aid providing, community, union, political, and ‘other’ associations), was coded ‘Yes’ (0), no participation (report of ‘No’ or ‘Don’t know’) was coded ‘No’ (1); and presence of children (No [1], Yes [0]). A response of ‘Yes’ to living alone, use tobacco, and inappropriate alcohol use (the latter defined as a positive answer to any of the following questions: 1. Has your family, your friends, your physician or your priest ever commented or suggested that you were drinking too much? 2. Have you ever tried to stop drinking but been unable to do so? 3. Have you ever had trouble at work or school because of alcohol, such as drinking or missing work? 4. Have you ever been involved in fights or arrested for being drunk? 5. Has it ever seemed to you that you were drinking too much?), was coded 1, and ‘No’ was coded 0.

**Physical health**. Self-rated health was assessed by asking participants “How would you rate your health?” Response was on a 5-point scale, dichotomized as impaired (fair, bad, very bad) vs. non-impaired (good, very good).

Dependence in activities of daily living (ADL) was assessed by response to each of the following five items: Do you need help (a) performing household activities (cleaning, maintenance, meal preparation), (b) taking medications, (c) with personal hygiene (bathing, combing hair, dressing, cutting nails), (d) feeding yourself, and (e) with mobility (sitting down, getting up, lying down, walking, going up stairs)? Response categories of “don’t know” and “no response” were rare and were recoded conservatively to “no” (need no help), and coded as 0; response of “yes” (need help) was coded as 1. Responses were summed (possible range 0–5), and the summed score dichotomized as yes (1) (≥1 activity for which help was needed) vs. no (0) (no help needed for any activity).

Enquiry into health conditions for which treatment had been sought in the previous six months included hypertension, heart problems, stroke, varicosities, diabetes, arthritis, back problems, osteoporosis, bronchitis, pneumonia, urinary infection, renal problems, dermatologic problems, headache (in the previous week), gastro-intestinal problems and cancer.

**Sensory impairments**. *Vision* was assessed by asking participants “How would you rate your visual ability?” Response was on a 6-point scale, dichotomized as impaired (blind, very bad, bad) vs. non-impaired (fair, good, very good).

*Hearing* was assessed by asking participants “How would you rate your hearing ability?” Response was on a 5-point scale, dichotomized as impaired (deaf, very hard to hear, hard to hear) vs. non-impaired (minimal or no difficulty hearing).

**Depression**. The presence of depression within the previous 30 days was determined by the six-item Short Psychiatric Evaluation Schedule (Short-SPES), validated for the older Brazilian population [22]. Each interviewer-administered question required a yes/no answer from the respondent. The total score (potential scoring range 0–6) was categorized as depression (score ≥4) vs non-depression (scores 0–3). A study of inter-rater reliability showed complete scoring agreement between examiners. Performance on the Short-SPES is not affected by sex, age, marital status, income, education, or minority status [22].

**Service use**. Use of health care was defined as self-report of any outpatient visit in the previous 6 months (yes/no), and any hospitalization within the previous 12 months (yes/no). If hospitalized, we asked whether there had been one or more than one hospitalization.

### Statistical analysis

Descriptive statistics (frequencies, χ^2^ tests) were used to characterize the sample and to test the bivariate relationships between physical inactivity and the variables of interest. The distribution of some variables was skewed, hence we decided to keep them dichotomized in order to get a balanced distribution. Adjusted logistic regression was used to study possible correlates of physical inactivity using three models: Model 1 included sociodemographic characteristics, Model 2 added social ties and health behaviors, and Model 3 further controlled for self-rated health, ADL, health conditions and depression. Separate logistic regression analyses were run to examine the association between physical inactivity and outpatient visits, hospitalization, and number of hospitalizations, first with an unadjusted model, and then with a model that adjusted for age, gender, ethnicity and socioeconomic status. These variables were selected because previous work suggested that they are associated both with the likelihood of physical inactivity as well as with health service use. Multivariate significance tests were performed using Wald χ^2^ tests. Statistical significance was evaluated using 2-tailed design-based tests. To control for Type I error inflation we adopted a more conservative p-value for significance: P<0.01. Analyses were carried out using SPSS version 20.0.

## Results

Of the 6,924 participants, 4,316 (62%) identified themselves as physically inactive. Table 1 gives the sociodemographic and clinical characteristics for the total sample and by physical inactivity status. In bivariate analyses, physical inactivity was more likely in women, increased with age (age 60–64: 58.2%; age ≥80: 72.8%), higher with lower education and income, and for those of Afro-Brazilian ethnicity (73% Afro-Brazilians; 61% White). Inactivity was also associated with no longer being married, being unemployed, and with no social activity participation. It was associated with impaired self-rated health, dependence in ADL, and heart problems, varicosities, arthritis, back problems, bronchitis, pneumonia, headache, sensory impairment, and depression (all met the criterion of P<0.01). Physically inactive participants were less likely to report an outpatient visit in the previous six months, but more likely to report hospitalization (but only one) in the previous12 months.

**Table 1:** Sample characteristics by physical inactivity, sociodemographic variables, social and health behavior, medical conditions and use of services (N = 6924)^1^.

		Physical Inactivity		
	Total sample	Inactive	Active	χ ^2^
	N (6924) N (%)	(N = 4316) N (%)	(N = 2608) N (%)	**p value**
***Sociodemographic characteristics***				
**Gender**				
Female	4572 (66.0)	3030 (66.3)	1542 (37.3)	.001
Male	2352 (34.0)	1286 (54.7)	1066 (45.3)	
**Age**				
60–64	1861 (26.9)	1084 (58.2)	777 (41.8)	.001
65–69	2077 (30.0)	1253 (60.3)	824 (39.7)	
70–74	1062 (15.3)	639 (60.2)	423 (39.8)	
75–79	1207 (17.4)	818 (67.8)	389 (32.2)	
80+	717 (10.4)	522 (72.8)	195 (27.2)	
**Education**				
<4 years	4565 (66.1)	3080 (67.5)	1485 (32.5)	.001
≥4 years	2339 (33.9)	1220 (52.2)	1119 (47.8)	
**Income**				
Low income	4310 (64.1)	2890 (67.1)	1420 (32.9)	.001
Higher income	2411 (36.0)	1285 (53.3)	1126 (46.7)	
**Ethnicity**				
Caucasians	5826 (84.2)	3567 (61.2)	2259 (38.8)	.001
Afro-Brazilian	472 (6.8)	346 (73.3)	126 (26.7)	
Other	624 (9.0)	402 (64.4)	222 (35.6)	
***Social ties and health behaviors***				
**Marital status**				
Married	3149 (45.5)	1806 (57.4)	1343 (42.6)	.001
Never married	468 (6.8)	307 (65.6)	161 (34.4)	
No longer married	3305 (47.7)	2201 (66.6)	1104 (33.4)	
**Children (Yes)**	6466 (93.6)	4032 (62.4)	2434 (37.6)	.814
**Live alone**	1052 (15.2)	655 (62.3)	397 (37.7)	.962
**Employed (no)**	5972 (86.4)	3796 (63.6)	2176 (36.4)	.001
**Use tobacco (Yes)**	1299 (18.8)	839 (64.6)	460 (35.4)	.063
**Alcohol (inappropriate) (Yes)**	734 (10.6)	457 (62.3)	277 (37.7)	.966
**Participate in social activities (yes)**	2733 (39.5)	1458 (53.3)	1275(46.7)	.001
***Self-rated health*, *activities of daily living*, *and health conditions***				
**Self rated health (impaired)**	4392 (63.5)	2942(67.0)	1450(33.0)	.001
**ADL (problem)**	2718 (39.3)	1883 (69.3)	835 (30.7)	.001
**Hypertension**	3404 (49.2)	2145 (63.0)	1259 (37.0)	.257
**Heart problems**	1955 (28.2)	1287 (65.8)	668 (34.2)	.001
**Stroke**	251 (3.6)	173 (68.9)	78 (31.1)	.028
**Varicose veins**	1197 (17.3)	786 (65.7)	411 (34.3)	.009
**Diabetes**	765 (11.0)	498 (65.1)	267 (34.9)	.094
**Arthritis**	2997 (43.3)	1969 (65.7)	1028 (34.3)	.001
**Back problem**	2993 (43.2)	1919 (64.1)	1074 (35.9)	.007
**Osteoporosis**	1046 (15.1)	680 (65.0)	366(35.0)	.053
**Bronchitis**	1917 (27.7)	1248 (65.1)	669 (34.9)	.003
**Pneumonia**	452 (6.5)	320 (70.8)	132 (29.2)	.001
**Urinary infection**	1219 (17.6)	796 (65.3)	423 (34.7)	.019
**Renal problems**	896 (12.9)	580 (64.7)	316 (35.3)	.112
**Dermatologic problem**	724 (10.5)	441 (60.9)	283 (39.1)	.404
**Headache**	2243 (32.4)	1488(66.3)	755 (33.7)	.001
**Gastrointestinal problem**	1270 (18.3)	791 (62.3)	479 (37.7)	.967
**Cancer**	94 (1.4)	68 (72.3)	26 (27.7)	.044
**Visual impairment**	1681 (24.3)	1198 (71.3)	483 (28.7)	.001
**Hearing impairment**	1063 (15.4)	717 (67.5)	346 (32.5)	.001
**Depression**	1453 (21.0)	1052 (72.4)	401 (27.6)	.001
***Health service use***				
**Out-patient visits**	4970 (72.1)	3044 (61.2)	1926 (38.8)	.005
**Any hospitalization**	1397 (20.2)	969 (69.4)	428 (30.6)	.001
**If any hospitalization, ≥2 hospitalizations**	427 (30.6)	304 (71.2)	123 (28.8)	.325

^1^ Information missing for some variables.

### Adjusted association between physical inactivity and sociodemographic variables (Table 2, model 1)

Physical inactivity was significantly related to all sociodemographic variables. In particular, women, older persons, those with less education, lower income, and of Afro-Brazilian ethnicity had increased odds of being inactive than their reference groups (respectively by 48%, 37%-70% depending on age category, 67%, 37%, and 49%).

**Table 2:** Logistic regression.

	Model 1				Model 2				Model 3			
	P	OR^1^	99% CI^2^		P	OR	99% CI		P	OR	99% CI	
			Lower	Upper			Lower	Upper			Lower	**Upper**
***Sociodemographic characteristics***												
Sex (female)	.001	1.478	1.281	1.706	.001	1.533	1.288	1.824	.001	1.458	1.216	1.749
Age category (y) (Reference = 60–64)												
65–69	.212	1.088	.914	1.294	.255	1.082	.906	1.293	.273	1.080	.902	1.292
70–74	.681	1.034	.839	1.275	.791	1.023	.823	1.270	.935	.993	.797	1.238
75–79	.001	1.374	1.116	1.690	.001	1.361	1.094	1.692	.002	1.308	1.047	1.635
80+	.001	1.695	1.306	2.200	.001	1.595	1.210	2.101	.001	1.459	1.096	1.943
Education (<4yrs)	.001	1.666	1.441	1.927	.001	1.586	1.368	1.840	.001	1.457	1.252	1.696
Income (low)	.001	1.374	1.186	1.592	.001	1.338	1.149	1.559	.001	1.249	1.068	1.460
Ethnicity (Reference = White)												
Afro-Brazilian	.001	1.490	1.118	1.986	.001	1.456	1.087	1.950	.001	1.470	1.094	1.975
Other	.683	1.038	.820	1.315	.715	1.035	.814	1.315	.916	1.010	.792	1.288
***Social ties and health behaviors***												
Marital status (Reference = Married)												
Never married					.381	1.118	.805	1.553	.318	1.137	.816	1.584
No longer married					.042	1.141	.966	1.348	.058	1.132	.957	1.340
Children (yes)					.524	1.082	.788	1.485	.714	1.047	.760	1.441
Live alone					.045	.854	.697	1.046	.070	.866	.705	1.062
Employed (no)					.615	1.042	.846	1.283	.762	.976	.790	1.205
Use tobacco (yes)					.029	1.168	.972	1.404	.068	1.142	.947	1.379
Alcohol inappropriate (yes)					.119	1.155	.910	1.465	.392	1.084	.851	1.379
Participate in social activities (no)					.001	1.767	1.541	2.028	.001	1.736	1.510	1.996
***Self-rated health*, *activities of daily living*, *and health conditions***												
Self-rated health (impaired)									.001	1.374	1.171	1.613
ADL (problem)									.001	1.242	1.066	1.447
Hypertension									.043	.886	.759	1.034
Heart									.293	.932	.784	1.108
Stroke									.623	1.078	.727	1.598
Varicose veins									.741	1.024	.849	1.237
Diabetes									.334	1.094	.861	1.389
Arthritis									.160	1.088	.932	1.270
Back problem									.520	.962	.824	1.123
Osteoporosis									.397	.935	.763	1.147
Bronchitis									.972	1.002	.851	1.180
Pneumonia									.057	1.250	.925	1.690
Urinary infection									.460	.945	.777	1.150
Renal problems									.469	.939	.751	1.174
Dermatologic problem									.122	.874	.698	1.094
Headache									.626	.970	.827	1.138
Gastrointestinal problem									.035	.861	.717	1.034
Cancer									.145	1.429	.761	2.685
Visual impairment									.001	1.277	1.073	1.518
Hearing impairment									.675	1.033	.845	1.264
Depression									.001	1.281	1.048	1.566
Logistic regression χ2				318.4				457.2				566.9
Degrees of freedom				9				17				38
P value				0.001				0.001				0.001
Pseudo R2				0.06				0.09				0.11

Odds of physical inactivity adjusting for sociodemographic characteristics (Model 1); and social ties and health behaviors (Model 2); and self-rated health, activities of daily living, and health conditions (Model 3) ^3^

^1^OR = odds ratio

^2^CI = confidence interval.

### Adjusted association between physical inactivity and sociodemographic variables, social ties and health behaviors (Table 2, model 2)

All sociodemographic associations were slightly modified but remained significant. Absence of involvement in social activities was the only new variable significantly associated with physical inactivity (OR 1.77).

### Adjusted association between physical inactivity and sociodemographic variables, social ties, health behaviors and health conditions (Table 2, model 3)

Odds ratios remained significant for all variables examined previously. With respect to measures of health and specific health conditions, physical inactivity was significantly associated only with impaired self-rated health (OR 1.37), dependence in ADL (OR 1.24), visual impairment (OR 1.28), and depression (OR 1.28). Association with other health conditions did not meet criteria for significance. Alternate analyses (not shown), including only self-rated health and ADL (i.e., excluding health conditions), and including specific health conditions but not self-rated health and ADL, modified little the associations already found.

### Physical inactivity and use of health services (Table 3)

In unadjusted analyses, physical inactivity was associated with increased odds of hospitalization (but not of multiple hospitalizations) in the previous 12 months (OR 1.47). Statistical significance for reduced odds of an outpatient visit in the previous 6 months was just missed. After adjusting for demographic characteristics, the odds of an outpatient visit in the previous 6 months was reduced significantly by 19%. Association with any hospitalization in the previous 12 months remained positive, with increased odds of 41%, but there was no association with multiple hospitalizations.

**Table 3:** Unadjusted and adjusted odds ratios of the association between physical inactivity and outpatient visits, hospitalization and number of hospitalizations.

	Unadjusted OR^2^ (99% CI^3^)	Adjusted^1^ OR (99% CI)
Any outpatient visit in past 6 months	0.87 (0.75–1.01)	0.81 (0.69–0.94)***
Any hospitalization in past 12 months	1.47 (1.24–1.74)***	1.41 (1.18–1.67)***
One hospitalization vs ≥2 hospitalizations	1.14 (0.82–1.59)	1.11 (0.79–1.55)

^1^ Sociodemographic characteristics (gender, age, education, income, ethnicity) controlled

^2^OR = odds ratio

^3^CI = confidence interval.

*** p<0.001

## Discussion

Our investigation showed that physically inactive seniors is very frequent particularly among Afro-Brazilians. Furthermore it is associated with adverse sociodemographic characteristics; lack of social interaction; and poor self-rated health, ADL, vision, and depression; although not with other health conditions. Self-care may be neglected, resulting in hospitalization.

In this analysis of a large state-wide sample of community residents age 60 and over from southern Brazil, 62% (66.3% of the women, 54.7% of the men) reported that they had not engaged in specific physical activity in the prior three months. This prevalence is higher than some other findings in Brazil for comparable age persons (40.7% in Florianópolis [16], 44.6% for men and 43.2% for women age 60–69, 57.0% for men and 69.1% for women age ≥70 years in Pelotas [23], 51% in Rio Claro [17], 56.5% in capitals of Brazil [24], 58% South and Northeast Brazil [15]), but lower than others (71.6% in São Paulo [19], 79.5% in Bambui [25], 87.2% in São Paulo [26]). Prevalence is lower than in urban areas in European Community countries (80% [14]), but similar to that for the U.S. population aged 65 years and over (62.2% [3;27]).

Internationally, despite some similarities across regions, heterogeneity in the prevalence of physical activity is nevertheless substantial. Hallal et al. (2012) [3], report that adults aged 60 years or older from Southeast Asia are not only more active than same age persons from all other regions, but also than young adults (aged 15–29 years) in the Americas, eastern Mediterranean, Europe, and the western Pacific. Follow up studies have indicated that physical inactivity is increasing rapidly world-wide, but particularly in China and Brazil, which have the two highest absolute and relative rates of increase in physical inactivity [4]. This is not surprising given that, as Ng et al. point out [4], in the past few decades the Chinese and Brazilians have been moving away from agriculture to large urban areas focusing on manufacturing and services. The high prevalence of physical inactivity among older adults may be explained in part by increased automation, use of motorized transport, and growth in media technologies (e.g., television, internet) [28]. If this tendency continues we can expect developing countries to reach the high level of physical inactivity of developed countries rapidly [29].

In agreement with others, we found that physical inactivity increased with age [15,18,19,23,30–33]. It has been suggested that increased inactivity with age is strongly associated with an underlying biologically driven aging process [34].

Consistent with previous studies [19–23], elderly with lower education and income were at greater risk of physical inactivity than were people with higher education and income. This may in part be attributable to differences in local resources (gymnasiums, safe sidewalks, safe neighborhoods), personal issues (fear of falling, lack of company), and access to television [17]. Unfortunately, we did not gather information in these areas.

We found a higher prevalence of physical inactivity among Afro-Brazilians, consistent with earlier studies showing Afro-Brazilians at higher risk than whites for physical inactivity [19]. A similar situation has been found for African Americans [10]. For both groups, this may be attributable to their average lower socioeconomic status, which is generally associated with poorer health [35]. One study, however, found a 25% higher physical inactivity rate in whites, but the sample was age 20 years and over [23]. A separate fully adjusted analysis of the Afro-Brazilians in the sample (data not shown), found that only one variable—absence of involvement in social activities—was significantly associated with physical inactivity in this group. It is possible that in our sample, the variability in this group, and its size, are too limited to permit identification of risk factors for physical inactivity.

A fully adjusted analysis indicated that absence of involvement in social activities was highly associated with physical inactivity. It is unclear whether this was attributable to personality, to environmental factors, or to incapacity or death of family and friends, but it is an area that merits investigation in future studies. Neither smoking nor alcohol use was significantly associated with physical inactivity. This may reflect a survival effect.

Consistent with earlier reports [19], unadjusted analysis found physical inactivity to be significantly associated with several clinical disorders, including heart problems, varicosities, arthritis, back problems, bronchitis, pneumonia, headache, sensory impairments and depression, but since this is a cross-sectional study, it is unclear whether we are looking at cause or consequence. To better understand the association between physical inactivity and health disorders, we controlled for sociodemographic characteristics, health behaviors and social behaviors, and for multiple morbidities. So doing indicated that only poor self-rated health, problems performing ADL, poor vision and depression remained significantly associated with physical inactivity. Self-rated health provides information beyond that provided by individual health conditions, is predictive of health service use, and of death [36]. ADL indicates the impact on everyday functioning of all conditions present, including those not specifically measured. Thus here both, independent of each other, indicate that physical inactivity is associated with poor current health. Our data cannot tell us what the outcome of intervening at this stage is likely to be, but studies that have examined exercise interventions suggest that mobility can be improved and health service use decreased [37].

Of the specific health conditions, only two conditions—visual impairment and depression—remained significantly associated with physical inactivity. The specific factors underlying associations between physical inactivity and visual impairment are not necessarily the same as the specific factors underlying associations between physical inactivity and depression. The association with depression is not unexpected, since diminished interest or pleasure, lack of energy and psychomotor retardation are hallmarks of this disorder. While visual impairment need not inhibit physical activity (consider the paralympic games), the absence of social contacts which could reduce the dangers associated with getting around when vision is poor, and the likely lack of training in safe performance when vision deteriorates in older age (as compared to training for visually impaired youth), may discourage activity.

The absence of an association between physical inactivity and specific health conditions in adjusted analyses appears to be explained by socioeconomic status. This suggests that environmental factors may be involved (as indicated above), or that non-leisure physical activity (occupation, household, or transportation activities), may leave little time for planned leisure activity, which has been found to have a more desirable effect on health [10].

In a recent study examining the association of physical inactivity with chronic diseases, Dogra [38] found, in a fully controlled analysis, that low levels of physical activity in older adults may be attributed to poor self-rated health itself, regardless of the presence of respiratory, musculoskeletal, or other chronic conditions. In other words, it is possible that chronic disease is not the reason for lower levels of physical activity observed in these groups, but rather, self-rated health. The results of the present study provide further support that chronic or infectious diseases should not be used to classify older adults as physically inactive [39]—here we found no association between chronic conditions (such as diabetes, heart disease, hypertension) or infectious diseases (pneumonia, urinary infection) and physical inactivity. It appears that some older adults with chronic conditions are successful at overcoming disease-associated activity obstacles, but others are not. Perhaps the subjective perception of health status plays a more important role in the physical inactivity levels of older adults than the simple presence of disease *per se*.

### Health services utilization

Our findings on use of health services were somewhat unexpected. In an adjusted analysis, physically inactive older participants were 19% less likely than those who were physically active to report any outpatient visit in the past 6 months. In contrast, they were 41% more likely to use be hospitalized in the previous 12 months (but not more likely to have multiple hospitalizations). This may reflect personal neglect by the physically inactive, or greater obstacles accessing regular outpatient care, for instance, common outpatient medications are paid for out-of-pocket (which may be difficult when income is low), but medications when hospitalized are covered [40]. Outpatient care neglect may result in an increased odds of hospitalization. Canadian findings indicate that physical inactivity was strongly associated with statistically significant increases in hospitalization, length of stay and healthcare visits, together with higher concomitant costs to the healthcare system [41].

Physical inactivity involves multiple, complex behavior processes [19–23], such that over time individuals may adopt a lethargic attitude to health care, reinforced by obstacles to health service use [40,41]. Despite investigatory efforts, little is known as to whether these factors affect receipt of health services among those with the disorders reported here, and under the circumstances under which they live.

### Strengths and Limitations

The strength and novelty of the current study is that we could take into consideration a wide range of confounders that could influence associations with physical inactivity. These included sociodemographic characteristics, health behaviors and social ties, alternative measures of health, a wide array of health conditions, and also depression. Other strengths of our study include the focus on older people (for whom information is needed), the representativeness of the sample, the relatively large number of participants, and the high response rate.

This study has certain limitations. Owing to the cross-sectional design, causality cannot be determined. In addition, we sampled only community residents, excluding persons in hospitals or nursing homes, where prevalence of physical inactivity is likely to be higher, however, the number in these settings is likely to be small. Due to socioeconomic and cultural disparities across geographic areas in Brazil the findings may not be generalizable beyond the area under investigation. Standard physical activity questionnaires, such as the International (or Global) Physical Activity Questionnaire [42], were not yet available at the time. The information on physical inactivity is based on self-report to a single item, and as is common in epidemiological surveys, did not use objective methods. It reflects absence of planned physical activity, but does not indicate intensity of physical activity, or physical activity in other areas. Since planned exercise is considered desirable, our approach may have resulted in over-reporting of physical activity because of social desirability, and so underestimate the prevalence of physical inactivity [43]. Our analyses may therefore be unduly conservative. Health conditions were self-reported, but self report has been found to provide valid assessments of health conditions [44]. While the diagnosis of depression was not based on the “gold standard” of a clinical interview, the Short-SPES is a valid and reliable tool for identification of depression in Brazilian community resident elderly [22]. The data were not available to discuss the major cultural, social, physical, and economic barriers that need to be addressed if behavior change is to be promoted in favor of increased physical activity or to discourage inactivity. While the data were gathered long ago, and the emphasis, through different medias mostly towards adolescents and adults, to increase physical activity have spread out, more recent information have shown similar figures than ours suggesting that the numbers found in this study can represent the current situation [15].

In summary, this study has shown that physical inactivity among older adults in the southern part of Brazil is highly prevalent, particularly among Afro-Brazilians. It is associated with low income and education; lack of social interaction; and poor self-rated health, ADL, vision, and depression; although not with other health conditions. Self-care may be neglected, resulting in hospitalization. Special attempts need to be made to target this population in order to encourage physical activity. Additional resources may be needed to provide the means whereby physical activity will be more accessible to and feasible for older persons with these kinds of problems. Recent studies and reviews indicate that several physical activity interventions have been identified as promising for future research and implementation [45]. Efforts to address active leisure will require taking safety, economic, personal, psychological, cultural, and social barriers into account [46]. Emphasizing physical activity among the elderly requires creativity in developing acceptable, relevant, culturally meaningful activities (e.g., dancing, typical local sports, tai chi, etc), and studies to ascertain outcomes and cost-effectiveness. Findings from the present study may help the public, health care providers, and those who determine public policy better address the issues of active lifestyle among the older population with the aim of improving health and well-being, and reducing health care costs.
